# Color Stability of Fermented Mare’s Milk and a Fermented Beverage from Cow’s Milk Adapted to Mare’s Milk Composition

**DOI:** 10.3390/foods9020217

**Published:** 2020-02-19

**Authors:** Joanna Teichert, Dorota Cais-Sokolińska, Romualda Danków, Jan Pikul, Sylwia Chudy, Paulina Bierzuńska, Łukasz K. Kaczyński

**Affiliations:** Department of Dairy Products Quality, Faculty of Food Science and Nutrition, Poznań University of Life Sciences, ul. Wojska Polskiego 31, 60-624 Poznań, Poland; cais@up.poznan.pl (D.C.-S.); dankow@up.poznan.pl (R.D.); japi@up.poznan.pl (J.P.); sylwia.chudy@up.poznan.pl (S.C.); paulina.bierzunska@up.poznan.pl (P.B.); lukasz.kaczynski@up.poznan.pl (Ł.K.K.)

**Keywords:** mare’s milk, fermented beverages, color

## Abstract

Color is important for the consumer when making a purchase decision. Mare’s milk and, thus, fermented mare’s milk is little known to consumers. Thus, it is worth presenting research showing the extent of color change during the production and storage of mare’s milk. Herein, we examined the range of color changes in mare’s milk and cow’s milks adapted to mare’s milk composition. These samples were further fermented and stored for 3 weeks at 5 ± 1 °C. Starter cultures containing *Streptococcus thermophilus* and *Lactobacillus delbrueckii* subsp. *bulgaricus* were used for fermentation. Mare’s milk reached the required pH of 4.5 during fermentation faster (255 min) than cow’s milk (300 min). After fermentation, mare’s milk compared to cow’s milk and adapted cow’s milk had lower titratable acidity (0.75%) and firmness (145. 6 |(g∙s)|). The water holding capacity (95.6%) and number of *Lactobacillus* (7.71 log CFU/mL) and *Streptocococcus* (7.20 log CFU/mL) in mare’s and other’s milks were the same. Mare’s milk was furthest from the ideal white (WI) color, with its chrome (C*) being 1.5-times larger than cow’s milk. However, fermented mare’s milk was darker than the fermented adapted milk and cow’s milk by 36% and 58%, respectively. Storage caused a decrease in the WI, C*, and yellowness index (YI). The fermented mare’s milk color stability during production and storage was less than that of fermented cow’s milk. After 3 weeks storage, it was observed that the titratable acidity increased to 1.05%, and the pH decreased to 4.3 in fermented mare’s milk. The water holding capacity decreased but was still higher compared to fermented cow’s milk.

## 1. Introduction

Cow’s milk is one of the most common nutritional sources for humans. The dairy industry produces a vast array of innovative dairy products, which are quickly distributed to the market. One of the motivators for this quick distribution is product expiration and consumer demand. Furthermore, nowadays, consumers require information regarding bioactive compound content and their role in milk nutrition. The modern consumer seeks dairy products that have greater nutritional value to complement their diet and nutritional needs. At the same time, the product needs to be sensorially attractive. Therefore, there is increased interest in products made from milk that are not mass-produced. The answer to these needs can be mare’s milk. The basic chemical composition and bioactive compound content in mare’s milk are different from cow’s milk, but are very similar to human milk [1,2]. Therefore, current research focuses on creating milk similar to mare’s milk and then combining it with the milk of other species [3,4]. Mare’s milk possesses immunoglobulins, lactoferrin, and lysozymes that show high biological activity, which benefit the human body [2,5]. The low fat content of mare’s milk makes it an ideal low-calorie alternative for people on reduced calorie diets [6,7]. Hence, mare’s milk is a source of high biologically active ingredients, which is a desirable way to influence the physiological processes occurring in the human body [8]. The pro-health value and digestibility of mare’s milk increases as a result of fermentation [9,10]. However, it should be noted that fermentation leads to physical and chemical changes in the raw material, such as changes in appearance, firmness, syneresis, and color.

Herein, we examined the change in color of fermented mare’s milk, which is one of the most important distinguishing features of a consumer’s product assessment [11]. Color influences product attractiveness and purchase decisions. In general, the amount of mare’s milk on the market is small, especially fermented mare’s milk. Unfortunately, consumers adhere to a standard color index for milk. Moreover, comparative studies or reports analyzing such colors are unavailable. Therefore, the observed color changes that occur during the parameterization of mare’s milk will contribute to product characterization. Furthermore, we examined the extent that the modification of cow’s milk had on its basic chemical composition and, in turn, its color in order to produce a raw material comparable to that of mare’s milk. This was achieved using the CIELab color measurement system, which has been applied in previous studies [12]. Also, we calculated the whiteness index (WI), yellowness index (YI), and chrome (C*), allowing them to be implemented as tools for quality control evaluation and the processing of mare’s milk.

Our hypothesis was that the color of fermented mare’s milk differs from the color of modified cow’s milk due to alterations in basic composition and that mare’s milk is stable during refrigeration storage and accepted by consumers.

## 2. Materials and Methods 

### 2.1. Mare’s and Cow’s Raw Milk Samples 

Mare’s milk was collected from Polish Coldblood mares over a 4 to 5 months lactation period, reared on an equine dairy farm in the Wielkopolska region (Western Poland). Mares were between 5 and 8 years of age with live weights between 607 and 840 kg. The mares were mechanically milked from both teats of the udder once a day in the evening after 3 h of physical separation from their foals for 12 days. Cow’s milk came from Polish Holstein-Friesian cows. Samples (20 L each) of bulk milk from mares and cows were collected according to ISO 707 [13].

### 2.2. Cow’s Milk Modification

The basic composition of cow’s milk was modified in order to make it similar to mare’s milk. This was conducted on a semi-technical scale. Whole cow’s milk was centrifuged at 40 °C (Bactofuge disc separator, type: D3187M, Alfa Laval, Richmond, VA, USA). The skimmed milk was subjected to two-fold microfiltration using a two-module system of ceramic membranes with a modified filtering layer (Isoflux, Tami Industrie, Nyons, France; Ø = 25 mm, length 1178 mm). The aim of the first stage of microfiltration was to reduce the total number of bacteria present in raw cow’s milk. This process was carried out using membranes with a pore diameter of 1.4 μm, flow velocity of 5.5 m/s, and transmembrane pressure of 0.2 to 1.0 bar. The obtained filtrate was subjected to further microfiltration using 0.14 μm pore diameter membranes at a flow velocity of 5.4 m/s and a transmembrane pressure between 0.2 to 1.5 bar. As a result of this process, whey proteins (permeate) were separated from casein (retentate). Permeate was thickened 1.7-times using a reverse osmosis, model RO3-type membrane with a 0.001 μm pore diameter (Sepro, Carlsbad, CA, USA). The flow velocity of the permeate was 1.3 m/s. The pressure before and after the diaphragm was 31 and 32 bar, respectively. This resulted in a concentrate with increased whey protein and lactose content similar to that of mare’s milk, which was supplemented with fat content up to the level of 14 g/kg. Then it was homogenized in a homogenizer (APV 1000 Albertslund, Denmark) with a pressure of 152 bar at 70–75 °C and cooled to 4 ± 1 °C.

### 2.3. Compositional and Physicochemical Analysis of Milk

The basic chemical composition was determined using DairySpec FT (Bentley Instruments, Chaska, MN, USA). The pH of milk was analyzed using a pH-meter model CP-505 (Elmetron, Zabrze, Poland) equipped with an electrode EPS-1 (Elmetron, Zabrze, Poland). The freezing point of the milk was determined according to ISO 5764 [14] using an advanced model 4D3 cryoscope with a 3LH700 thermistor probe (Advanced Instruments Inc., Norwood, MA, USA). The values of dynamic viscosity of the unfermented milk were determined utilizing the method described by Cais-Sokolińska et al. [15] using a Höppler KF10 viscosity meter by RheoTec Messtechnik GmbH (Ottendorf, Germany). 

### 2.4. Milk Fermentation Process

Mare’s, cow’s, and modified cow’s milks were pasteurized (72 °C, 15 s) in a milk pasteurizer (P 300 EL, Plevnik, Dobrova, Slovenia) and cooled to 43 ± 1 °C. In the production of beverages, the starter cultures *Streptococcus thermophilus* and *Lactobacillus delbrueckii* subsp. *bulgaricus* were used. Commercially available Lyofast Y 480 F, from Sacco (Cadorago, Italy) was added at 30 units/100 L processed milk. Fermentation ran at 43 °C until pH 4.5 was obtained. The product was poured into PS (polystyrene) containers with a 150 g product capacity and then cooled to 5 ± 1 °C. The product was tested 48 h after the end of the fermentation process (0 weeks) and after 3 weeks of storage at 5 ± 1 °C. The process ran on a pilot plant scale (*n* = 12).

### 2.5. Fermentation Parameters

The pH was measured using a CP-502 pH-meter (Elmetron, Zabrze, Poland) equipped with an ESAgP-301W combination electrode (Eurosensor, Gliwice, Poland) composed of glass and saturated silver chloride half-cells. The pH was automatically recorded at 15-min intervals. The maximum acidification rate (V_m_) was calculated from the pH curves, according to the equation V_m_ = (ΔpH/Δt), and expressed in absolute values (unit pH/min). The maximum rate, V_m_, along with the time at which the maximum acidification rate was observed, Tm (min), and the time, T_e_ (min), at which a pH of 4.5 was reached were the measured responses that characterized the fermentation [16]. The titratable acidity values were expressed in Soxhlet-Henkel degrees (°SH, 1 °SH = 0.0225 lactic acid %). The sample was titrated with standardized 0.25 N NaOH using 1 mL 2% phenolphthalein as an indicator, giving an end-point of a faint pink color.

### 2.6. Color Analysis 

A milk sample before and after fermentation was placed in an optical glass cuvette (2/96G10, Starna Scientific Company Ltd., Ilford, UK) with dimensions of 28 mm × 16 mm × 40 mm and a volume of 7.2 mL. The measurement was performed using a D65 light source whose continuous spectrum in the visible range was similar to that of natural light and at a 10° observation angle using an X-Rite SP-60 camera (Grandville, MI, USA) equipped with a spherical geometry (diffusive), with measurement chamber possessing a DRS-811 ceramic insert. The camera was calibrated based on the black and white SP-62-162 reference standards (X-Rite). The color was analyzed based on CIELab: L* (lightness), a* (−green/+red color), and b* (−blue/+yellow color) [17]. The whiteness index (WI), chrome (C*), and yellowness index (YI) were calculated using the following equations [12]:

C* = [(Δa*)^2^ + (Δb*)^2^]^0.5^, (1)

WI = [(ΔL*)^2^ + (Δa*)^2^ + (Δb*)^2^]^0.5^, and (2)

YI = 142.86b*/L*,(3)

where the calculations assume that L* = 100, a* = 0 and b* = 0.

### 2.7. Lactic Acid Bacteria

The isolation and determination of *Lactobacillus* lactic acid bacteria were performed on MRS, i.e., according to de Man, Rogosa, and Sharpe, agar substrate no. 110660 (Merck KgaA, Darmstadt, Germany) [18]. The substrate (68.2 g/L) had a pH of 5.7 at 25 °C following dissolution and autoclaving (15 min at 121 °C). It was infected with the test material by the cast-iron method. The incubation was carried out at 37 ± 1 °C for 72 h under anaerobic conditions in a binder thermostat model WTB (Tuttlingen, Germany).

The isolation and determination of the number of *Streptococcus* lactic acid bacteria were performed on M-17 agar medium no. P-0220 (BTL, Łódź, Poland), as proposed by Terzaghi and Sandine for breeding and determining the number of lactic streptococci in milk and milk products [19]. The substrate (57.3 g/L) had a pH of 7.0 ± 0.2 at 25 °C following dissolution and autoclaving (15 min at 121 °C). It was infected with the test material by the cast-iron method. The incubation was carried out at 35 ± 1 °C for 24–48 h under anaerobic conditions in a WTB binder thermostat (Tuttlingen, Germany).

### 2.8. Determination of Water Holding Capacity

The water holding capacity (WHC) of a sample is defined as its ability to hold all or part of its own water. The WHC of the samples was determined using a slightly modified centrifugation method [20]. The sample (30 g) was centrifuged (model 260; MPW MED Instruments, Warsaw, Poland) under a relative centrifugal force (RCF) of 10,732 g and rotor angle of 30° (RPM 10 062 g) at 4 °C for 15 min. The supernatant was collected and weighed and the WHC was calculated according to the following equation:

WHC (%) = (1 − W_1_/W_2_) · 100, (4)

where W_1_ is the weight in grams of the supernatant after centrifugation and W_2_ is the weight of the sample in grams.

### 2.9. Gel Firmness

The firmness of the fermented samples were determined using reverse extrusion in a TA-XTplus texture meter from Stable Micro Systems (Surrey, UK). The A/BE attachment with a compression disc (Ø = 35 mm) was used. A sample was placed inside a cylinder with an internal diameter of Ø = 50 mm (75% filling). The measurement conditions were a distance of 30 mm, pre-test 1.0 mm/s, and post-test 10.0 mm/s. Results were recorded in Texture Exponent E32 version 4.0.9.0 software (Godalming, Surrey, UK).

### 2.10. Acceptability of Color and Appearance 

Fermented milks and beverages from the adapted cow’s milk were evaluated in the sensory test. Consumers (*n* = 92; 51 female, 41 male; aged 24 to 57; M_age_ = 34.77, SD = 9.87; Caucasian race) were asked to indicate how much they liked or disliked each product on a 9-point hedonic scale (9 = extremely like; 1 = extremely dislike) based on color and appearance. Randomized, refrigerated (10 °C) samples of 10 mL were served in clear glasses with a volume of 50 mL and were marked with three-digit random numbers [21].

### 2.11. Statistical Evaluation

Verification of the statistical hypotheses was accomplished by adopting an α = 0.05 level of significance using the Statistica data analysis software, version 10 (StatSoft, Tulsa, OK, USA). The influence of the composition and storage time on the milk fermented products was evaluated by two-way analysis of variance (one-way ANOVA) followed by Tukey’s HSD post-hoc test for multiple comparisons. The skewness, which is a measure of asymmetry, was used to assess normality distribution.

## 3. Results

### 3.1. Characteristics of Beverages from Mare’s Milk and Cow’s Milk Adapted to Mare’s Milk Composition

Gross composition and physicochemical properties of mare’s milk are presented in Table 1. The solid non-fat content of 84.1 g/kg was consistent with values obtained by other reports [7,22]. Fat content (13.2 g/kg) corresponded to the values obtained by Caroprese et al. [23]. The protein content was similar to that indicated by Potočnik et al. [24] for the same breed. The dominant ingredient in mare’s milk is lactose (65.0 g/kg). There was one-third more lactose in mare’s milk than in cow’s milk (*p* < 0.05), which influenced the dry matter content. The obtained results showed that the adapted cow’s milk compared to mare’s milk only differed in pH (*p* > 0.05). Mare’s milk had a higher pH than cow’s milk due to lower casein and phosphate concentrations [25]. The parameters of mare’s milk significantly differed (*p* < 0.05) from whole cow’s milk except for freezing point.

### 3.2. Color of Mare’s and Other Milks

Mare’s milk was the furthest from the ideal white standard (WI = 21.5, Table 2) and differed from other milk samples (*p* < 0.05). The yellowness index (YI) and chrome also showed the extent to which mare’s milk differs from cow’s milk (*p* < 0.05). Adaptation of cow’s milk to mare’s milk in terms of gross composition did not cause color similarities. The chrome (C*) of mare’s milk (C* = 4.2) was about 1.5-times greater than cow’s milk (C* = 2.5, *p* < 0.05). Furthermore, the analysis of lightness value showed that mare’s milk is darker compared to cow’s milk and even adapted cow’s milk (L* = 78.9, *p* < 0.05). The reason for such significant differences is due to variations between inter alia, casein, and fat contents. Mare’s milk contains 21.4% protein (50% casein, 38.8% whey protein) [26]. The casein content in mare’s milk is lower than in cow’s milk and is the reason why mare’s milk is included in so-called albumin milks [23]. The white color of the milk is mainly due to light dispersion by colloidal molecules of the casein-calcium complex, the presence of insoluble orthophosphate (V) tri-calcium Ca_3_(PO_4_)_2_, and weakly soluble calcium hydrogenorthophosphate (V) calcium CaHPO_4_ [27].

### 3.3. Fermentation Parameters and Selected Physicochemical and Microbiological Attributes of Fermented Mare’s and Other Milks

Mare’s milk was characterized by the highest fermentation dynamics (V_m_) (Table 3). Within 105 min, mare’s milk had quickly reached the required pH 4.5. This was 45 minutes shorter than cow’s milk fermentation time. Titratable acidity in mare’s milk was lower than in cow’s milk and cow’s milk after modification (*p* < 0.05). However, after 3 weeks, the titratable acidity of mare’s milk increased and was larger than cow’s and adapted cow’s milks (*p* < 0.05). The number of lactic acid bacteria in fermented mare’s and others’ milks was the same and did not change due to storage (*p* > 0.05). After storage, it was shown that cow’s milk and adapted cow’s milk have a lower water holding capacity than immediately after production. In addition, the WHC of fermented mare’s milk was larger than cow’s milk and its adapted milk. The centrifugation time and centrifugal force allowed to separate casein from the supernatant. The casein precipitated was most noticeable in fermented cow’s milk after storage. Our research did not get a significant separation of casein from serum in fermented mare’s milk. The supernatant, after the spinning of the fermented mare’s milk, was not as clear as it was in cow’s milk. The casein precipitated from that milk was not very succinct. Cow’s milk and cow’s milk adapted to mare’s milk composition were characterized by a higher firmness, as measured with a texture meter, than fermented mare’s milk. This trend was consistent even after 3 weeks. The firmness of fermented mare’s milk was 2.7-times smaller than fermented cow’s milk (*p* > 0.05).

### 3.4. Color of Fermented Mare’s and Others’ Milk

Regardless of refrigeration storage time, statistically significant differences in WI were observed in all analyzed fermented milks (*p* < 0.05) (Table 4). The largest chrome (C*) was observed immediately after production in fermented modified cow’s milk. The YI of fermented mare’s milk was 1.5-times higher than that of fermented cow’s milk (*p* < 0.05). The 3 week period of refrigeration storage decreased WI, C*, and YI (*p* < 0.05).

**Figure 1:**
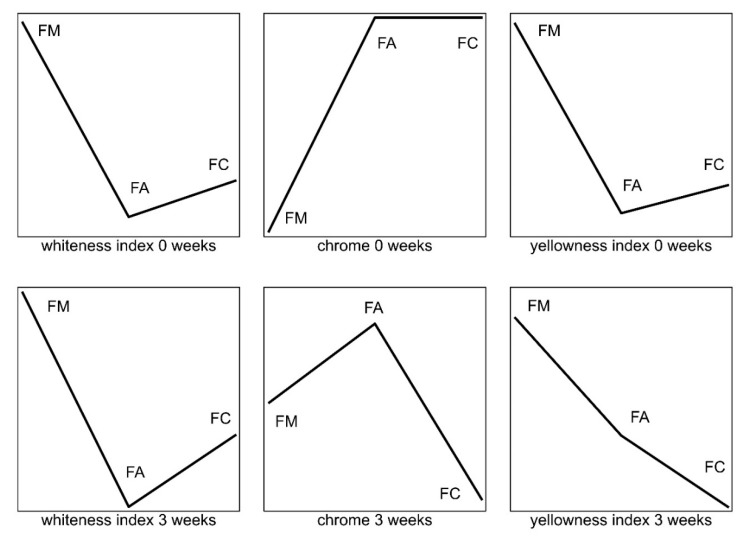
Graphic representation of changes in color indices due to storage (3 weeks) of fermented mare’s milk and beverages from cow’s milk adapted to mare’s milk composition. FM: fermented mare’s milk; FA: beverages from cow’s milk adapted to mare’s milk; FC: fermented cow’s milk.

The values of the calculated color indices are presented in the form of multi-dimensional lines using the exploratory technique (Figure 1). This allowed for the identification of interactive links between fermented mare’s milk and beverages from cow’s milk modified to the composition of mare’s milk. The relative values of the selected variables of each fermented milk were represented by the height of the successive points of refraction on the line above the baseline. The course and position of the graphical objects, such as lines, helped assess the direction of changes in the WI, C*, and YI of the samples due to storage.

### 3.5. Overall Acceptability of Color

Almost a third of consumers dislike the color and appearance of fermented mare’s milk after a production time of 0 weeks (Table 5). Even more so, after 3 weeks, almost all consumers (90%) disliked its color and appearance, with the remainder (10%) being indecisive. The reasons for rejection and lack of acceptance were the transparency and blue/grey color. Dislike responses were about 1% at 0 weeks and the same after 3 weeks.

## 4. Discussion

Color is an important factor influencing consumer assessment of product quality [28]. The L* value in fermented mare’s milk was lower than that of fermented adapted milk and increased during refrigeration storage. Our research has shown that the value of L* for beverages from cow’s milk also increased during storage (Table 4). Our study showed that beverages from modified cow’s milk and cow’s fermented milk are 1.36- and 1.58-times lighter than fermented mare’s milk. Cais-Sokolińska and Pikul [29] showed similar results for cow’s milk. Increasing the acidity of the milk causes the destabilization of the casein complex and the protein fractions are then transformed from a micellar state into a state of dispersion. This change in the ionic system contributes to the reduction in brightness. Remeuf et al. [30] stated that a high WHC in yogurt with a greater whey protein content could be related to the higher solvation of the micellar system and to a more branched yogurt microstructure. So as a result, yogurt is less susceptible to losing water during centrifugation. Increasing the whey protein-to-casein ratio in the milk base improves the WHC of the yogurt [31]. Use of caseinate to fortify milk gave yogurts with lower WHCs [32]. Sodini et al. [31] during the centrifugation (1290× *g*, 20 min, 8 °C) of yogurt (12.5% total solids in it 1.4% fat) received a WHC of 80%. The WHC of yogurt increases when the protein/total solids ratio in the milk base is increased by partially replacing skim milk powder with whey protein concentrate. Authors noticed that yogurt can show a WHC close to that of skim milk powder-enriched yogurt with a lower total solids content (13.4% vs. 16%). Additionally, Berber et al. [33] showed that whey protein denaturation enhanced gelling properties and, hence, the WHC with adequate heat treatment. Protein denaturation was responsible for the increase in the water holding capacity of yogurt.

Miloradovic et al. [34] examined the color change of cheese packed under a modified atmosphere. A significant effect was found in salt concentration and packaging conditions on the b* value, but it was impossible to visually determine the total difference in color among cheese variants. They also noticed that the measurement of color indicates the significance of external factors, while the sample remains acceptable. Consumers gave higher rates to fermented beverages from cow’s milk adapted to mare’s milk composition 8% (vs. 29.4% in the case of mare’s fermented milk). Consumers were not satisfied at the beginning and, after 3 weeks, this increased to 21% (vs. 90.2% in case of mare’s fermented milk). Consumers liked the color and appearance of most fermented cow’s milk (Table 5, Figure 1). Bierzuńska et al. [12] also analyzed the color of yogurts and studied the effects of adding whey protein concentrate after the yoghurt polymerization process. The addition of unpolymerized whey proteins increased the YI, C*, and WI compared to the yogurt control. Importantly, on the basis of the color measurement, it is possible to optimize and select conditions for the technological process. The fundamental white difference between mare’s milk and cow’s milk results from a different proportion of proteins (casein and whey proteins). In our research, the content of casein in mare’s milk was 12.9 g/kg and 9.8 g/kg whey protein, whereas in cow’s milk it was 27.6 and 6.7 g/kg, respectively. There is no data in the literature about the influence of serum proteins on milk color. Most studies concern the influence of whey protein as a powder concentrate of fermented cow’s milk. The study by Delikanli and Ozcan [35] showed that whey protein significantly affected the color (L*, a*, and b*) of yogurt. The L* parameter of natural yogurt and whey protein-based yogurt was 90.2 and 95.1, respectively, and the b* parameter, determining the yellow color, was 12.3 and 13.8, respectively. Gonzalez-Martinez et al. [36] described a yellowish color that developed in yogurt when whey powder was added. Supavititpatana et al. [37] described the change in yogurt color as a result of the addition of whey powder.

There is a lack of information about the color of milk and dairy products. It is worth using color measurement to evaluate changes in the quality of products, especially during storage. Further research may show the measurement of color alterations is equivalent to quality changes, which can be faster to measure than the consumer’s eye.

## 5. Conclusions

The color of mare’s milk differs from cow’s milk. Measured color parameters (L*, a*, b*) and calculated indices (WI, C*, YI) showed that mare’s milk is less white, although the saturation of the C* indicator was higher. Research has shown that the lack of acceptance of the color and appearance of fermented mare’s milk, especially after storage, is a serious problem to product overall acceptability. Consumers are accustomed to the characteristic whiteness of cow’s milk. However, the change in the composition of adapted cow’s milk was noticeable to the consumer. When comparing cow’s milk adapted to mare’s milk composition and cow’s milk, it was more distant from ideal white, more saturated (higher C* value), and had a higher YI value. The color stability, indicated by changes in the YI, of fermented mare’s milk was higher than that of fermented cow’s milk. These results will contribute to comprehensive technical and technological conditions for the process of mare’s milk development. Research into the choice of technologies that will increase consumer acceptance of mare’s milk should be further examined.

## Figures and Tables

**Table 1:** Gross composition and physicochemical characteristics of mare’s milk, cow’s milk adapted to mare’s milk composition, and cow’s milk.

Parameters	Mare’s Milk	Adapted Cow’s Milk	Cow’s Milk
Solid non-fat (g/kg)	84.1 ± 0.7 ^a^	86.6 ± 0.3 ^b^	91.2 ± 0.9 ^c^
Fat (g/kg)	13.2 ± 1.1 ^a^	14.0 ± 0.9 ^a^	41.2 ± 0.6 ^b^
Protein (g/kg)	22.9 ± 0.8 ^a^	22.7 ± 2.4 ^a^	34.4 ± 0.8 ^b^
Casein (g/kg)	12.9 ± 0.7 ^a^	12.8 ± 1.8 ^a^	27.6 ± 0.9 ^b^
Whey protein (g/kg)	9.8 ± 0.4 ^b^	9.8 ± 1.2 ^b^	6.7 ± 0.6 ^a^
Lactose (g/kg)	65.0 ± 1.0 ^c^	63.3 ± 1 ^b^	48.8 ± 0.2 ^a^
pH	6.92 ± 0.03 ^c^	6.56 ± 0.04 ^a^	6.64 ± 0.03 ^b^
Freezing point (°C)	−0.551 ± 0.002 ^a^	−0.535 ± 0.017 ^a^	−0.541 ± 0.034 ^a^
Viscosity (mPas)	3.05 ± 0.02 ^a^	3.12 ± 0.06 ^a^	4.31 ± 0.26 ^b^
Density (in 20°C, kg/m^3^)	1.037 ± 1 ^b^	1.036 ± 2 ^b^	1.028 ± 2 ^a^

Values represent mean ± standard deviation (*n* = 12). Different small letters in the superscript in rows indicate statistically significant differences at the level α = 0.05.

**Table 2:** Assessment of the color of mare’s milk, cow’s milk adapted to mare’s milk composition, and cow’s milk.

Parameters	Mare’s Milk	Adapted Cow’s Milk	Cow’s Milk
WI	21.49 ± 1.03 ^c^	17.82 ± 1.41 ^b^	8.92 ± 1.04 ^a^
C*	4.17 ± 0.19 ^c^	3.17 ± 0.24 ^b^	2.54 ± 0.16 ^a^
YI	6.60 ± 0.40 ^c^	4.33 ± 0.47 ^b^	3.41 ± 0.16 ^a^
L*	78.92 ± 1.04 ^a^	82.47 ± 1.46 ^b^	91.46 ± 1.10 ^c^

WI, whiteness index; C*, chrome; YI, yellowness index; L*, lightness value. Values represent mean ± standard deviation (*n* = 12). Different superscript small letters in the superscript in rows indicate statistically significant differences at the level α = 0.05.

**Table 3:** Acidity, syneresis, firmness, and lactic acid bacteria of fermented beverages from mare’s milk, cow’s milk adapted to mare’s milk composition, and cow’s milk.

Parameters	Fermented Milk Beverage					
	Mare’s	Adapted Cow’s	Cow’s	Mare’s	Adapted Cow’s	Cow’s
	0 Weeks			3 Weeks		
pH	4.51 ± 0.06 ^b^	4.50 ± 0.04 ^b^	4.50 ± 0.05 ^b^	4.33 ± 0.06 ^a^	4.34 ± 0.02 ^a^	4.38 ± 0.04 ^a^
V_m_ (unit pH/min)	0.0131	0.0084	0.0080			
T_m_ (min)	105	150	210			
T_e_ (min)	255	285	300			
Titratable acidity (%)	0.76 ± 0.04 ^a^	0.84 ± 0.03 ^b^	0.86 ± 0.04 ^b^	1.05 ± 0.15 ^c^	0.86 ± 0.03 ^b^	0.87 ± 0.05 ^b^
WHC (%)	95.6 ± 1.9 ^c^	96.0 ± 1.3 ^c^	96.7 ± 0.8 ^c^	91.5 ± 1.5 ^b^	80.0 ± 2.9 ^a^	78.6 ± 1.7 ^a^
Firmness |(g∙s)|	145.6 ± 26.5 ^a^	279.2 ± 55.2 ^b^	410.0 ± 37.6 ^c^	149.9 ± 41.6 ^a^	275.3 ± 61.1 ^b^	399.0 ± 30.4 ^c^
*Lactobacillus* (log CFU/mL)	7.71 ± 0.37 ^a^	7.83 ± 0.36 ^a^	7.89 ± 0.48 ^a^	7.28 ± 0.44 ^a^	7.18 ± 0.94 ^a^	6.76 ± 0.69 ^a^
*Streptococcus* (log CFU/mL)	7.20 ± 0.75 ^a^	7.27 ± 0.83 ^a^	7.20 ± 0.74 ^a^	7.33 ± 0.61 ^a^	7.55 ± 0.67 ^a^	7.01 ± 0.61 ^a^

V_m_, maximum acidification rate; T_m_, time at which; V_m_ is reached; T_e_, time to reach pH 4.5. WHC, water holding capacity; CFU, colony-forming unit. Values represent mean ± standard deviation (*n* = 12). Different superscript small letters in the superscript in rows indicate statistically significant differences at the level α = 0.05.

**Table 4:** Assessment of the color of fermented mare’s milk, fermented cow’s milk adapted to mare’s milk composition and fermented cow’s milk.

Fermented Product	Storage (w)	WI	C*	YI	L*
Mare’s milk	0	45.16 ± 0.35 ^f^	5.54 ± 0.24 ^bc^	13.28 ± 0.68 ^d^	55.18 ± 0.34 ^a^
	3	42.16 ± 0.56 ^e^	5.11 ± 0.49 ^b^	10.98 ± 1.21 ^c^	58.15 ± 0.57 ^a^
Adapted cow’s milk	0	25.95 ± 0.58 ^d^	6.07 ± 0.41 ^d^	10.46 ± 0.75 ^c^	74.78 ± 0.65 ^b^
	3	23.19 ± 0.76 ^c^	5.75 ± 0.36 ^cd^	9.25 ± 0.79 ^b^	77.54 ± 0.80 ^b^
Cow’s milk	0	14.04 ± 0.92 ^b^	5.65 ± 0.37 ^cd^	8.57 ± 0.62 ^b^	87.16 ± 1.04 ^c^
	3	14.92 ± 0.62 ^a^	4.14 ± 0.30 ^a^	5.96 ± 0.59 ^a^	85.67 ± 0.63 ^c^

w, weeks. Values represent mean ± standard deviation (*n* = 12). Different small letters in the superscript in columns indicate statistically significant differences at the level α = 0.05.

**Table 5:** Sensory acceptability of color and appearance of fermented beverages from mare’s milk, cow’s milk adapted to mare’s milk composition, and cow’s milk.

Hedonic Scale (1–9)		Fermented Beverage from Milk					
		Mare’s	Adapted Cow’s	Cow’s	Mare’s	Adapted Cow’s	Cow’s
		0 Weeks			3 Weeks		
9	Like extremely	3	11	39		0	11
8	Like very much	5	18	19		3	25
7	Like moderately	2	25	21		19	28
6	Like slightly	22	22	7		39	18
5	Neither like nor dislike	33	9	5	9	12	9
4	Dislike slightly	27	4	1	10	19	1
3	Dislike moderately	0	3	0	23	0	
2	Dislike very much	0	0	0	36	0	
1	Dislike extremely	0	0	0	14	0	
Skewness		0.97	0.49	1.41	1.22	1.35	0.62
p-value		0.01	0.27	0.02	0.05	0.02	0.08
SD		13.23	9.48	13.50	12.56	13.49	11.16
CV		129.39	92.78	132.05	122.85	131.95	109.13
Dislike responses (%)		29.35	7.61	1.09	90.22	20.65	1.09

SD, standard deviation; CV, coefficient of variation.
